# INTRA- AND INTER-RATER RELIABILITY OF A BIOPHOTOGRAMMETRIC ASSESSMENT PROTOCOL FOR PRETERM INFANTS

**DOI:** 10.1590/1984-0462/2021/39/2020034

**Published:** 2020-12-07

**Authors:** Juliana Vieira Campos, Mariana Alves Moreno, Ricardo de Bastos Silva, Jessica Neves Quirino da Silva, Milena Ferreira de Carvalho, Rayssa Christina Abreu dos Santos, Rodrigo Tosta Peres, Rosana da Silva Santos, Halina Cidrini Ferreira

**Affiliations:** a Universidade Federal do Rio de Janeiro, Rio de Janeiro, RJ, Brazil.; b Centro Federal de Educação Tecnológica Celso Suckow da Fonseca, Rio de Janeiro, RJ, Brazil.

**Keywords:** Infant, premature, Physical therapy modalities, Photogrammetry, Recém-nascido prematuro, Modalidades de fisioterapia, Fotogrametria

## Abstract

**Objective:**

To measure the intra- and inter-rater reliability of a biophotogrammetric assessment protocol for thoracoabdominal motion in preterm infants.

**Methods:**

This is an analytical cross-sectional study. Footage of 40 preterm infants was made in two views (lateral and anterior). The babies were placed in the supine position, with retroverted pelvis and semiflexed knees. Acrylic markers were positioned on surgical tape in eight predetermined anatomical points. We analyzed 4 variables in lateral view and 11 in anterior view (angular and linear) (ImageJ^®^), divided into two stages: 1. same frames - three blinded evaluators analyzed frames previously selected by the main researcher (inter-rater analysis 1), reviewing these same frames after 15 days (intra-rater analysis 1); 2. different frames - each evaluator selected the frames from the original video and repeated the protocol (inter-rater analysis 2), with a review after 15 days (intra-rater analysis 2). In stage 2, we tested the reliability of the entire process, from image selection to the analysis of variables. Data agreement and reproducibility were obtained by the intraclass correlation coefficient (ICC).

**Results:**

Agreement was high, particularly in angular variables (ICC 0.82 to 0.99). Linear variables ranged between very good and excellent in analysis 1 (same frames: ICC 0.64 to 0.99) and analysis 2 (different frames: ICC 0.44 to 0.89).

**Conclusions:**

The present study suggests that the proposed protocol for the thoracoabdominal motion analysis of preterm neonates has high reliability.

## RESUMO

**Objetivo:**

Mensurar a confiabilidade intra e interexaminador de um protocolo de avaliação biofotogramétrica da mobilidade toracoabdominal de prematuros.

**Métodos:**

Estudo de caráter transversal e analítico. Incluíram-se filmagens de 40 prematuros em duas vistas (lateral e superior), realizadas em supino, pelve retrovertida e joelhos em semiflexão. Marcadores de acrílico foram posicionados sobre Micropores em oito pontos anatômicos predeterminados. Foram analisadas 4 variáveis na vista lateral e 11 na vista superior (angulares e lineares) (ImageJ^®^), divididas em duas etapas: (1-*Frames* iguais) análises de fotogramas previamente selecionados pela pesquisadora principal por três avaliadores cegos (análise interexaminador 1), com reanálise desses mesmos fotogramas após 15 dias (análise intraexaminador 1); (2-*Frames* diferentes) cada avaliador selecionou os fotogramas por meio do vídeo original e repetiu o protocolo (análise interexaminador 2), com reanálise após 15 dias (análise intraexaminador 2). Em (2), foi testada a confiabilidade de todo o processo de análise, desde a separação das imagens até a análise das variáveis. A concordância e reprodutibilidade dos dados foram obtidas pelo coeficiente de correlação intraclasse (CCI).

**Resultados:**

Houve concordância forte, com ênfase nas variáveis angulares (CCI [0,82 a 0,99]). As variáveis lineares apresentaram variação entre muito boa e excelente na análise 1 (*frames* iguais: CCI 0,64 a 0,99) e na análise 2 (*frames* diferentes: CCI entre 0,44 e 0,89).

**Conclusões:**

O presente estudo sugere forte confiabilidade do protocolo proposto para análise da movimentação toracoabdominal de neonatos prematuros.

## INTRODUCTION

The technical-scientific advances in prenatal care and life support after birth enable the survival of children born with increasingly lower gestational ages.^1^ However, neuropsychomotor and respiratory diseases and conditions may occur more often,^2^ ^,^ ^3^ making crucial the specialized monitoring of growth and development in this population from birth to adulthood.^4^ ^,^ ^5^ Prematurity-related complications are the main causes of neonatal death and the second leading cause of death in children up to five years of age, only after pneumonia.^6^

Preterm children present immaturity in a variety of organs and systems. As a result, prolonged periods of hospitalization, with a potential need for mechanical ventilation, oxygen therapy, and intensive resources, are frequent.^7^ ^,^ ^8^ ^,^ ^9^

Instruments that evaluate respiratory changes caused by prematurity are scarce in neonatal units, and the few tools available are more connected to experimental protocols, in addition to being expensive and/or invasive. Thus, the respiratory function assessment in care practice mainly depends on the subjective, personal, and little standardized interpretation of evaluators.^10^ ^,^ ^11^

The thoracic motion and pulmonary function assessments allow the situational diagnosis of the baby and the prevention of possible diseases. Single and multiple occlusion techniques, cirtometry,^12^ and inductance plethysmography^13^ are some of the methods used for these purposes. Some of these techniques are compatible with the adult and pediatric population, as is the case of cirtometry, but they are dysfunctional when applied to neonatology.^12^ ^,^ ^13^ Innovating and adapting methods to meet the needs of the neonatal population are relevant and necessary.

Photogrammetry is the science of measuring through photographs,^14^ including quantitative assessments of images and videos. It uses markers placed on anatomical reference points to measure different angles and distances.^15^ Since this tool has proven to be versatile and easy to adapt to different areas of medicine,^15^ it has gained relevance in several age groups, especially for evaluating the thoracic area.^14^ ^,^ ^15^

Nevertheless, we found no reliability and reproducibility measurements for the existing protocols, which restricts their bedside use.^14^ ^,^ ^15^ Besides, the variables still do not answer all questions that arise in clinical practice and are difficult to measure in daily routine.^14^ ^,^ ^15^ These considerations justify the creation of a new protocol with the performance of a reliability and reproducibility analysis to substantiate its informed and safe use in the preterm infant population. Moreover, we found no reports on easy-to-acquire angle and distance measurements, which are relevant for understanding the dynamics of breathing in neonatal units.

Considering the above, this study aimed at evaluating the intra- and inter-rater reliability of a new photogrammetric protocol to assess the thoracoabdominal motion in preterm infants.

## METHOD

This is an analytical cross-sectional study approved by the Research Ethics Committee of the Teaching Maternity Hospital of Universidade Federal do Rio de Janeiro, under the Certificate of Presentation for Ethical Consideration no. 47024515.4.0000.5275.

The present investigation included videos of children of both genders, born with less than 37 weeks of gestational age, who were in neonatal units, aged up to 90 days, clinically stable, and whose guardians allowed their participation in the research by signing the informed consent form. Prematurity was defined according to the World Health Organization report, which classifies extremely preterm infants as individuals born before 28 weeks of gestational age, very preterm infants as those born between 28 and less than 32 weeks of gestational age, and moderate to late preterm infants as the ones born between 32 and less than 37 weeks of gestational age.^16^

We excluded sedated and/or curarized newborns and infants, patients diagnosed with severe gastroesophageal reflux, and those who had congenital malformations. Additionally, we excluded babies with hemodynamic instability, using vasoactive amines, diagnosed with pulmonary hypertension, and who had any other condition that could interfere with clinical stability.

The sample calculation was obtained with the following Equation 1:

$$\frac{n=N.Z^{2}.p.(1-p)}{Z^{2}.p.\left(1-p\right)+e2.(N-1)}$$ (1)

We adopted a 5% sampling error and a 95% confidence level. The sample calculation was performed based on the total number of births in the teaching maternity hospital according to the last verification of the Live Birth Information System (2013), indicating an approximate sampling goal of 40 preterm infants, recruited by convenience.^17^

The profile data of newborns (name, date of birth, date of hospitalization, birth weight, weight on the day of data collection, and gestational age, gathered from their medical records) were checked, and their vital signs and pulse oxygen saturation were monitored throughout data collection and recorded at the beginning and end of the process. Alertness was measured by the Brazelton scale.^18^ Babies who presented a score up to 5 in the Brazelton scale advanced to the next stage.

Extra pillows and rolls were removed, leaving only those essential for the proper positioning of the newborn. Their clothes were removed, except for the disposable diaper. The newborn/infant was placed in the supine position with the head centralized, upper limbs alternating between free and restricted, semiflexed hips and knees, and pelvic anteversion.

A team member was chosen to stay with the newborn/infant, ensuring their permanence in this position and fulfilling their possible needs, such as: non-nutritive sucking, touch-pressure, and temporary changes in position.

Four small squares of Micropore^®^ surgical tape were placed on their skin (for protection) at the following anatomical points: glabella, right acromion, left acromion, and xiphoid process of the sternum. Another four small squares of the same surgical tape were positioned on the sides of the chest: two following the line of the xiphoid marker to the right and left side of the chest, and the other two placed bilaterally at the level of the last ribs (4 cm from the central xiphoid process marker). After placing the Micropore^®^ pieces, the colored acrylic markers were glued to the protective tapes in the eight points mentioned above.

Lateral-view images were acquired by a camera (Nikon^®^, COOLPIX S6200, Tokyo, Japan) attached to a tripod (Greika^®^, WT3716, São Paulo, Brazil) at the right side of the patient’s bed, adjusted so that the camera lens was parallel to the baby’s midline. The distance between the camera and the newborn was 31 cm when the baby was in the cradle and 44 cm when they were in the incubator.

The shooting lasted a minute, and, after the recording, the tripod (still with the camera attached) was repositioned above the bed to capture anterior-view images, also for a minute. The camera remained at a distance of 70 cm when the baby was in the cradle and 14 cm from the outer upper limit of the incubator when the baby was inside the equipment. These distances were predefined so that the image obtained would always include the outer edges of the bed in which the newborn was.

 After acquiring the images, the bed was reorganized, and the vital signs and oxygen saturation were collected again.

The videos recorded were transferred to the computer, organized into folders, and identified. The software Kinovea^®^ (Joan Charmant& Contributors, Bordeaux, France) was used to capture the frames (pictures of the video). Each frame was calibrated from pixels to centimeters by a reference point of known distance. The circular acrylic marker, with a linear diameter of 1 cm, was used in all images.

Four frames were selected from the videos of each patient, two in lateral view and two in anterior view (frames captured during maximum inspiration and maximum expiration found in each video) and analyzed by three blinded and independent raters (rater 1, rater 2, and rater 3).

**Figure 1:**
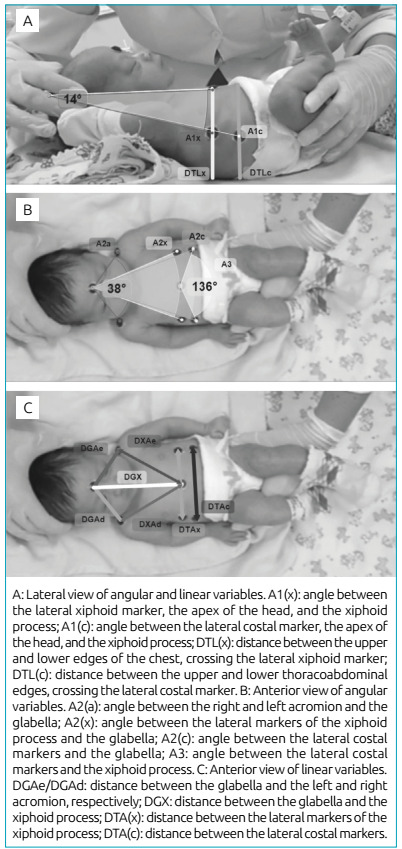
Presentation of the angles and distances measured.

The variables were quantified by the software ImageJ^®^ (Research Services Branch, National Institute of Mental Health, Bethesda, Maryland, United States) and are illustrated in Figure 1. Images were analyzed in two stages. In the first (stage 1), named same frames, three blinded raters analyzed the same frames (chosen by a single and different researcher) - inter-rater analysis (moment 1). These frames were reviewed 15 days after the first measurement - intra-rater analysis (moment 2).

Stage 2, named different frames, was carried out to verify the reliability of the full method, including frame selection, calibration, and analysis of variables, a process closer to how other researchers who wish to reproduce the protocol would perform future procedures. Thus, the blinded raters received the videos and conducted the whole process from the frame selection. Inter- and intra-rater analyses were also carried out, respecting the same 15 days between them.

Table 1 shows the study variables. All angular measurements were expressed as degrees (º), and linear measurements as centimeters (cm).

**Table 1:**
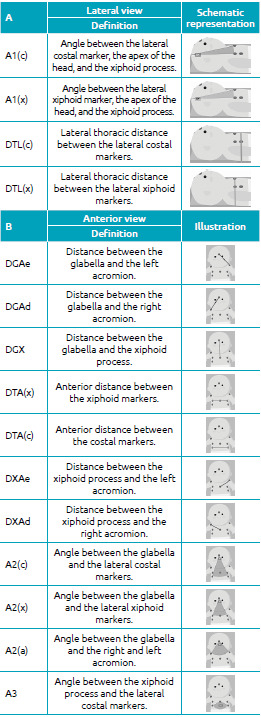
Study variables. A: lateral view B: anterior view.

Summary, organization, and description of the data set were performed. The data set received a descriptive treatment, with the calculation of measures of central tendency (mean) and dispersion (standard deviation). We used the intraclass correlation coefficient (ICC) with 95% confidence intervals to evaluate intra- and inter-rater reliability, as follows: excellent agreement (ICC>0.80); very good agreement (0.80≤ICC≤0.61); good agreement (0.60≤ICC≤0.41); fair agreement (0.40≤ICC≤0.21); and poor agreement (ICC<0.20).^17^ All procedures considered a 95% significance level (p<0.05).

## RESULTS

The study included 40 preterm newborns/infants, distributed^19^ in: 6 extremely preterm infants (15%), 21 very preterm infants (53%), and 13 moderate to late preterm infants (32%). The babies had a mean gestational age of 30±3 weeks and a birth weight of 1385±445 g.

As to the classification of birth weight x gestational age,^16^ we identified 28 (70%) infants considered adequate for the gestational age, and 12 (30%) regarded as small for the gestational age.

Regarding gender, the sample consisted of 23 male newborns/infants (57%) and 17 female ones (43%). At the time of data collection, the neonates were aged 28±21 days and weighed 1901±435 g.

During stage 1, same frames, each rater applied the protocol to 160 frames (40 frames during inspiration + 40 frames during expiration in each view, totaling 160 frames). The protocol was applied again after 15 days, totaling 2,400 analyses (considering all study variables). In stage 2, different frames, each rater applied the protocol from the frame selection, with 320 frames analyzed (80 during inspiration + 80 during expiration in each view, totaling 320 frames), and performed 4,800 assessments in the two moments of evaluation (considering all study variables).

Tables 2 to 4 present the thoracoabdominal motion data obtained from the analysis of anterior- and lateral-view images. Variable values were respectively attributed to each of the three raters. The two moments of analysis were indicated as moment 1 and moment 2. We found high ICC levels with respect to angular measurements, both in the intra-rater (same and different frames - Tables 3 and 4) and the inter-rater (same and different frames - Table 2) analyses, with values between 0.82 and 0.99, considered excellent agreement. Distance variables presented classifications between very good and excellent agreement in intra- and inter-rater analyses of the same frames (0.64 to 0.99). In intra- and inter-rater analyses of different frames, diameter variables showed good and very good agreement, with ICCs between 0.44 and 0.89.

**Table 2:** Intraclass correlation coefficient values of the inter-rater analysis followed by their confidence interval.

	Same frames		Different frames	
	Moment 1	Moment 2	Moment 1	Moment 2
A1(c)	0.98 (0.96; 0.99)	0.97 (0.95; 0.98)	0.94 (0.88; 0.97)	0.95 (0.92; 0.97)
A1(x)	0.98 (0.98; 0.99)	0.98 (0.97; 0.98)	0.96 (0.95; 0.98)	0.95 (0.93; 0.97)
DTL(c)	0.73 (0.63; 0.81)	0.63 (0.46; 0.75)	0.62 (0.45; 0.74)	0.52 (0.25; 0.70)
DTL(x)	0.73 (0.62; 0.82)	0.63 (0.41; 0.77)	0.63 (0.46; 0.76)	0.56 (0.36; 0.70)
DGAe	0.80 (0.56; 0.90)	0.87 (0.80; 0.92)	0.68 (0.58; 0.77)	0.59 (0.47; 0.70)
DGAd	0.84 (0.57; 0.93)	0.90 (0.85; 0.93)	0.70 (0.60; 0.78)	0.80 (0.71; 0.86)
DGX	0.74 (0.44; 0.86)	0.88 (0.83; 0.92)	0.65 (0.54; 0.75)	0.68 (0.54; 0.78)
DTA(x)	0.64 (0.31; 0.80)	0.85 (0.74; 0.91)	0.56 (0.43; 0.68)	0.73 (0.54; 0.83)
DTA(c)	0.70 (0.39; 0.84)	0.88 (0.78; 0.93)	0.59 (0.46; 0.69)	0.75 (0.59; 0.85)
DXAe	0.54 (0.29; 0.71)	0.76 (0.63; 0.84)	0.49 (0.36; 0.61)	0.63 (0.44; 0.76)
DXAd	0.52 (0.28; 0.69)	0.81 (0.70; 0.88)	0.47 (0.34; 0.60)	0.61 (0.46; 0.73)
A2(c)	0.99 (0.98; 0.99)	0.95 (0.93; 0.97)	0.87 (0.82; 0.91)	0.84 (0.78; 0.89)
A2(x)	0.98 (0.96; 0.99)	0.96 (0.95; 0.97)	0.88 (0.83; 0.92)	0.84 (0.78; 0.89)
A2(a)	0.99 (0.98; 0.99)	0.94 (0.91; 0.96)	0.89 (0.84; 0.92)	0.83 (0.76; 0.88)
A3	0.95 (0.93; 0.97)	0.91 (0.88; 0.94)	0.88 (0.83; 0.92)	0.88 (0.83; 0.91)

A1(x): angle between the lateral xiphoid marker, the apex of the head, and the xiphoid process; A1(c): angle between the lateral costal marker, the apex of the head, and the xiphoid process; DTL(x): distance between the upper and lower edges of the chest, crossing the lateral xiphoid marker; DTL(c): distance between the upper and lower thoracoabdominal edges, crossing the lateral costal marker. Anterior view of angular variables: A2(a): angle between the right and left acromion and the glabella; A2(x): angle between the lateral markers of the xiphoid process and the glabella; A2(c): angle between the lateral costal markers and the glabella; A3: angle between the lateral costal markers and the xiphoid process. Anterior view of linear variables: DGAe/DGAd: distance between the glabella and the left and right acromion, respectively; DGX: distance between the glabella and the xiphoid process; DTA(x): distance between the lateral markers of the xiphoid process; DTA(c): distance between the lateral costal markers. Moment 2: 15 days after analysis of moment 1.

**Table 3:** Intraclass correlation coefficient values of the same frame intra-rater analysis followed by their confidence intervals.

	Rater 1	Rater 2	Rater 3
A1(c)	0.99 (0.98; 0.99)	0.99 (0.98; 0.99)	0.99 (0.98; 0.99)
A1(x)	0.99 (0.98; 0.99)	0.99 (0.99; 1)	0.99 (0.98; 0.99)
DTL(c)	0.76 (0.65; 0.84)	0.90 (0.84; 0.93)	0.85 (0.74; 0.88)
DTL(x)	0.77 (0.66; 0.85)	0.92 (0.87; 0.94)	0.82 (0.78; 0.90)
DGAe	0.91 (0.86; 0.94)	0.95 (0.92; 0.97)	0.86 (0.79; 0.91)
DGAd	0.93 (0.89; 0.95)	0.96 (0.94; 0.97)	0.86 (0.79; 0.91)
DGX	0.86 (0.79; 0.91)	0.92 (0.87; 0.95)	0.84 (0.76; 0.89)
DTA(x)	0.80 (0.70; 0.86)	0.87 (0.81; 0.92)	0.82 (0.73; 0.88)
DTA(c)	0.85 (0.77; 0.90)	0.90 (0.85; 0.93)	0.86 (0.79; 0.91)
DXAe	0.70 (0.56; 0.79)	0.85 (0.77; 0.90)	0.68 (0.54; 0.78)
DXAd	0.71 (0.58; 0.80)	0.82 (0.73; 0.88)	0.70 (0.57; 0.80)
A2(c)	0.99 (0.99; 0.99)	0.99 (0.99; 0.99)	0.94 (0.91; 0.96)
A2(x)	0.99 (0.98; 0.99)	0.99 (0.99; 1)	0.95 (0.93; 0.97)
A2(a)	0.99 (0.98; 0.99)	0.99 (0.99; 1)	0.92 (0.88; 0.95)
A3	0.96 (0.94; 0.97)	0.98 (0.97; 0.99)	0.91 (0.86; 0.94)

Legend: A1(x): angle between the lateral xiphoid marker, the apex of the head, and the xiphoid process; A1(c): angle between the lateral costal marker, the apex of the head, and the xiphoid process; DTL(x): distance between the upper and lower edges of the chest, crossing the lateral xiphoid marker; DTL(c): distance between the upper and lower thoracoabdominal edges, crossing the lateral costal marker. Anterior view of angular variables: A2(a): angle between the right and left acromion and the glabella; A2(x): angle between the lateral markers of the xiphoid process and the glabella; A2(c): angle between the lateral costal markers and the glabella; A3: angle between the lateral costal markers and the xiphoid process. Anterior view of linear variables: DGAe/DGAd: distance between the glabella and the left and right acromion, respectively; DGX: distance between the glabella and the xiphoid process; DTA(x): distance between the lateral markers of the xiphoid process; DTA(c): distance between the lateral costal markers.

**Table 4:** Intraclass correlation coefficient values of different frame intra-rater analysis followed by their confidence intervals.

	Rater 1	Rater 2	Rater 3
A1(c)	0.97 (0.95; 0.98)	0.95 (0.93; 0.97)	0.99 (0.98; 0.99)
A1(x)	0.98 (0.96; 0.99)	0.96 (0.94; 0.98)	0.99 (0.99; 1)
DTL(c)	0.61 (0.45; 0.73)	0.79 (0.68; 0.86)	0.80 (0.70; 0.87)
DTL(x)	0.56 (0.39; 0.69)	0.78 (0.68; 0.86)	0.76 (0.65; 0.84)
DGAe	0.67 (0.53; 0.78)	0.56 (0.39; 0.70)	0.78 (0.68; 0.85)
DGAd	0.68 (0.54; 0.78)	0.77 (0.67; 0.85)	0.84 (0.75; 0.89)
DGX	0.64 (0.49; 0.75)	0.70 (0.57; 0.80)	0.78 (0.68; 0.85)
DTA(x)	0.48 (0.29; 0.63)	0.81 (0.72; 0.87)	0.82 (0.74; 0.88)
DTA(c)	0.58 (0.41; 0.71)	0.84 (0.75; 0.89)	0.85 (0.78; 0.90)
DXAe	0.57 (0.41; 0.70)	0.63 (0.47; 0.74)	0.65 (0.51; 0.76)
DXAd	0.54 (0.37; 0.68)	0.55 (0.38; 0.69)	0.67 (0.53; 0.77)
A2(c)	0.88 (0.81; 0.92)	0.83 (0.74; 0.88)	0.92 (0.88; 0.95)
A2(x)	0.89 (0.84; 0.93)	0.80 (0.70; 0.87)	0.93 (0.89; 0.95)
A2(a)	0.87 (0.80; 0.91)	0.84 (0.76; 0.89)	0.86 (0.79; 0.91)
A3	0.88 (0.82; 0.92)	0.85 (0.78; 0.90)	0.91 (0.86; 0.94)

Legend: A1(x): angle between the lateral xiphoid marker, the apex of the head, and the xiphoid process; A1(c): angle between the lateral costal marker, the apex of the head, and the xiphoid process; DTL(x): distance between the upper and lower edges of the chest, crossing the lateral xiphoid marker; DTL(c): distance between the upper and lower thoracoabdominal edges, crossing the lateral costal marker. Anterior view of angular variables: A2(a): angle between the right and left acromion and the glabella; A2(x): angle between the lateral markers of the xiphoid process and the glabella; A2(c): angle between the lateral costal markers and the glabella; A3: angle between the lateral costal markers and the xiphoid process. Anterior view of linear variables: DGAe/DGAd: distance between the glabella and the left and right acromion, respectively; DGX: distance between the glabella and the xiphoid process; DTA(x): distance between the lateral markers of the xiphoid process; DTA(c): distance between the lateral costal markers

## DISCUSSION

The results presented herein confirm that the photogrammetric protocol proposed has satisfactory intra- and inter-rater reliability in the preterm newborn/infant population. We found the highest ICCs in angular variables (ICC>0.81) when compared to linear variables. The protocol described is new since the variables measured have not been proposed by any other work and can provide relevant information about thoracoabdominal motion in different clinical situations of neonatal care.

Other protocols for the assessment of breathing in newborns and children using biophotogrammetry suggest measuring the thoracic and abdominal areas.^15^ ^,^ ^20^ ^,^ ^21^ ^,^ ^22^ ^,^ ^23^ The biophotogrammetric analysis of respiratory mechanics (*biofotogrametria para análise da mecânica respiratória* - BAMER) originated from the irregular quadrilateral model, described in several previous studies on respiratory kinematics,^24^ ^,^ ^25^ ^,^ ^26^ ^,^ ^27^ ^,^ ^28^ and proposes the establishment of transverse planes and the delimitation of the thoracic and abdominal compartments. Two studies included two new sub-compartments in each original compartment and obtained the thoracoabdominal edges drawn by straight lines on contours of anterior and posterior body surfaces.^26^ ^,^ ^28^

A later work conducted a biophotogrammetric chest analysis, through an adapted version of the BAMER model, in adults with dynamic hyperinflation after exercise, using the positive end-expiratory pressure. The author found results comparable to those obtained by more robust systems of respiratory kinematics and concluded that photogrammetry adds quantitative data relevant to respiratory monitoring.^15^

Another subsequent study performed a biophotogrammetric analysis of 19 asthmatic children with a mean age of 11 years based on the BAMER model adapted for the supine position in lateral view. The authors calculated the relative contributions of each sub-compartment compared to their original compartment and the chest wall. Thoracoabdominal motion was evaluated during isovolume maneuvers after maximum inspiration. The authors found a significant difference in the compartment and sub-compartment areas individually (p<0.001). The relative contributions compared to the chest wall were also significantly different (p<0.001). On the other hand, the ratios between sub-compartments and their original compartments showed no significant difference. The method proved to be effective in differentiating the movements of each area and identifying the more and less contributory regions to the respiratory movement of the compartment analyzed.^19^

This area measurement model and AutoCAD^®^ have been used to increase the evidence base related to different physical therapy techniques. In 20 full-term neonates, area measurements were taken before and after the increased expiratory flow technique, and no significant differences were found in their movements.^20^ Gomes et al. conducted a study with 40 full-term newborns, also measuring the thoracoabdominal compartment area, comparing the vibrocompression technique and the thoracoabdominal rebalancing. They detected no significant difference; however, the results were antagonistic between the techniques applied.^22^

The variables analyzed in this study to measure the thoracoabdominal motion of newborns and infants were not previously used and showed satisfactory intra- and inter-rater agreement. These measurements can translate the patients’ breathing dynamics cycle by cycle. In addition, they can quantify the results of movements that are empirical in clinical practice, such as variations in diameter and angle throughout the cycle (a negative variation between inspiration and expiration, for instance, can be understood as a possible paradoxical movement, in which the chest is drawn and does not expand as physiologically expected). The observation and evaluation of thoracic motion in neonatology are essentially subjective, and the lack of objective assessment parameters makes it difficult to carry out research and create follow-up routines that include these movements. We also underline that reliability measures for the neonatal population, which are crucial to know the reproducibility of protocols, are new and imperative before considering the implementation of these protocols in clinical assessment.

The relevance of this study is associated with the proposal of angular and linear variables that use key anatomical points as references for identifying chest retractions and distortions. The analyses are manually made, excluding the possibility of not identifying an inspiratory chest retraction, for example, which would not be objectively detected in the BAMER method.

The immediate consequences of preterm birth have a great impact on the motor and respiratory systems.^5^ However, studies assessing the late effects of prematurity also show frequent motor, cognitive, neurosensory, and respiratory changes. Garcia et al. associated preterm birth with thoracic musculoskeletal abnormalities in adolescents (10 to 15 years) and identified that adolescents born prematurely presented more evident static thoracic musculoskeletal abnormalities compared to the population of adolescents born at term.^3^ Given this context, developing assessment tools that can be ready to use in the near future is paramount in neonatal care. Late effects of prematurity have proven to be associated with very low and extremely low birth weight, in addition to prolonged mechanical ventilation during hospitalization in the neonatal unit.

Stick reported the possible significant relationships between intrauterine lung development, respiratory symptoms, and pulmonary function in adulthood. The author also mentioned the importance of knowing the interactions between developing pulmonary, genetic, and environmental factors for early diagnosis and the elaboration of new strategies to reduce the morbidity of chronic pulmonary diseases in the long term.^2^ Thus, constant evaluation and respiratory outpatient follow-up after hospital discharge for all individuals born prematurely become relevant to minimize immediate and late damages.

Concerning the technique proposed for protocol application, we emphasize that the anatomic points were carefully palpated for placement of the markers, with high methodological rigor to avoid errors, as well as measurement and analysis bias. Care with the neonate’s position, measurements for the placement of the camera, and data analysis were essential. Possible reproductions without such care may not properly translate the results.

Limitations for the performance of this work involve the routine of the intensive therapy environment as to the observance of the times of administration of diet, hygiene, and medication, always considering the minimum handling required to preserve the quality of life of the hospitalized baby. Occasionally, postponing data collection was necessary so that the routine of the newborn or infant would be disrupted as little as possible, which increased the time necessary to complete the protocol. On the other hand, the possibility of evaluating the thoracic motion in a non-invasive and highly reliable way allows the increasingly earlier screening of preterm infants, and, consequently, the prevention of a large part of undesired immediate and late respiratory outcomes. Another important limitation is the fact that photogrammetry uses 2D images, which can lead to parallax error, mainly when measuring distances with a lack of depth. This factor may explain the lower ICC in distance measurements compared to angular measurements. At any rate, the ICC levels are very good, which, besides not invalidating the measures, provide evaluation parameters for thoracic motion not available in neonatology.

Considering the above, the proposed protocol has good reliability and reproducibility and, therefore, can be used in the preterm infant population in the neonatal hospitalization environment, with the implementation of the full protocol, from frame selection to pure image analysis.
